# Psychedelic Experiences During the Early COVID-19 Pandemic: Findings From an International Online Survey

**DOI:** 10.3389/fpsyt.2021.732028

**Published:** 2021-11-04

**Authors:** Ricarda Evens, Simon Reiche, Roman M. Marek, Daa Un Moon, Rosa Elisa Groß, Amy Romanello, Dario Jalilzadeh Masah, Matteo Scicchitano Böckheler, Stefan Gutwinski, Christiane Montag, Inge Mick, Tomislav Majić

**Affiliations:** ^1^Research Group Psychotropic Substances, Psychiatric University Clinic at Hospital St. Hedwig | CCM, Charité—Universitätsmedizin Berlin, Corporate Member of Freie Universität Berlin and Humboldt-Universität zu Berlin, Berlin, Germany; ^2^Department of Psychiatry and Neurosciences | CCM, Berlin Institute of Health, Charité—Universitätsmedizin Berlin, Corporate Member of Freie Universität Berlin and Humboldt-Universität zu Berlin, Berlin, Germany; ^3^Interdisciplinary Research Group “The Future of Medicine: Good Health for All,” Berlin-Brandenburg Academy of Sciences and Humanities, Berlin, Germany; ^4^German Rheumatism Research Centre Berlin (DRFZ), Berlin, Germany; ^5^Berlin School of Mind and Brain, Humboldt-Universität zu Berlin, Berlin, Germany

**Keywords:** corona, pandemic (COVID-19), SARS-CoV-2, drug use, psychedelic, experience, survey, setting

## Abstract

**Introduction:** The current corona virus disease (COVID-19) pandemic has caused a serious global health crisis that has affected large parts of the public and private life worldwide, including the use of psychoactive substances. In this study, we investigated the effect of the COVID-19 pandemic on the use of serotonergic psychedelics, i.e., the settings in which people use psychedelics, the motives of usage, and the subjective quality of psychedelic experiences.

**Methods:** The study was part of an international, cross-sectional, internet-based survey (*N* = 5,049) available in five languages (English, German, Spanish, Italian, and Korean) carried out during the early phase of the pandemic from April to August 2020. Participants were asked to retrospectively rate settings and motives of psychedelic substance use before the pandemic and in the last 4 weeks during the pandemic, as well as changes in psychedelic experiences.

**Results:** Of *n* = 1,375 participants that reported the use psychedelics in 2019 or 2020, *n* = 642 (46.6%) also took psychedelics during the pandemic. During the pandemic, participants used psychedelics significantly less often in settings that were outside their home. Top motives to use psychedelics were comparable before and during the pandemic, but participants consumed less out of curiosity, to celebrate, or because friends took it, and more out of boredom. An increase in positively connoted, often pro-social experiences was observed. Two thirds of participants who used psychedelics during the pandemic claimed that psychedelics had helped them to deal better with the corona pandemic at least slightly.

**Discussion:** Changes in setting and motives were mostly in line with restrictions caused by control measures to contain the spread of the virus. The unexpected increase in positively connoted experiences possibly reflects a favorable interaction of environmental macro- and individual micro-contexts during the pandemic (e.g., by reducing the use in more uncontrolled recreational settings or by encouraging a strong self-selection of substance users due to the expectation of “bad trips”). Increased pro-social feelings under psychedelics might reflect a desire for social interactions in times of social distancing and pandemic-related stress and anxiety.

## Introduction

Beginning at the end of 2019 and reaching its first peak in most countries in early 2020, the current corona virus disease (COVID-19) pandemic has caused a serious global health crisis affecting large parts of public and private life worldwide (1). Profound changes in living conditions have resulted not only from the infectious disease caused by the new severe acute respiratory syndrome coronavirus type 2 (SARS-CoV-2), but also from the extensive measures implemented to contain the spread of the virus (2). Most countries have introduced measures restricting the freedom of movement, social contacts, and other aspects of public life (3). Those far-reaching changes in daily life have been associated with increased mental health problems, including symptoms of depression, anxiety, stress, and insomnia (4–6).

Many of these pandemic related measures have also affected drug use, drug supply, and drug markets (7). A large international online survey on drug use that was conducted during the early phase of the COVID-19 pandemic revealed that the frequency of drug use, during the pandemic compared to before the pandemic, varied across substances: While the consumption of stimulants more commonly used at parties and in other social contexts, like 3,4-methylenedioxymethamphetamine (MDMA, ecstasy) and cocaine, had declined, the use of tetrahydrocannabinol (THC)-containing cannabis products and prescription benzodiazepines had increased (8). It has been suggested that the latter changes in substance use might reflect a maladaptive coping strategy in the face of challenges imposed by this worldwide public health crisis (9, 10). As drug markets appeared to rapidly adapt, e.g., by shifting from face-to-face to online sales, local drug supply underwent constant changes during the pandemic (7). The impact of the COVID-19 pandemic on drug markets, however, varies greatly across countries, possibly depending on the respective measures implemented to contain the spread of SARS-CoV-2, and the extent to which those measures affect the pre-pandemic routes of production and trafficking (11).

In this context, serotonergic psychedelics represent a substance group for which relatively little is known about how it is affected by the pandemic. This group of psychoactive substances includes substances like lysergic acid diethylamide (LSD), psilocybin, and mescaline, which share psychedelic properties and serotonin (5-HT_2A_) receptor agonism as mechanism of action (12). The use of serotonergic psychedelics has been controversially discussed over the last decade. While it received considerable positive attention as a potential treatment tool for specific mental disorders, potential risks for mental health associated with psychedelic substance use have also been critically emphasized (13–15). Part of this discrepancy may be resolved by emphasizing the role of extra-pharmacological, contextual factors that are known to strongly affect the quality of psychedelic experiences (16). Those contextual factors include individual characteristics of the user (“set”) and aspects of the environment in which the experience is taking place (“setting”) (17, 18). The specific characteristics of the terms “set” and “setting” are not uniformly defined but represent broad concepts according to which non-pharmacological context factors can be classified. The substance users' “set” has been described to include stable individual characteristics like personality traits, as well as transient features of mood, motivation, intentions, or personal preparation for use (19). The term “setting” encompasses all characteristics of the substance users' “physical, social, and cultural environment” [(19), p. 1]. Unfavorable contextual conditions, i.e., unfavorable set and setting variables, have previously been associated with an increased likelihood of adverse reactions or challenging experiences during psychedelic substance use (13, 20).

While the significance of context has frequently been described, few studies have systematically explored different types of contexts. Most contemporary clinical research trials carefully design the context to increase the likelihood of experiences that are considered as helpful, e.g., through patient selection, detailed preparation of participants, provision of a safe environment, and social support (21). Investigating conditions that might be unfavorable for participants appears, on the other hand, to be highly unethical. While these interventions usually address the immediate environment during substance use (or “micro-context”), it has been emphasized that the context is not only characterized by aspects of the micro-context but also by “the wider social, cultural, and economic milieu” in which the substance use is embedded (“macro-context”) [(22), p. 5]. Given the temporal stability of social, cultural, and economic environments, an exploration of intraindividual changes concerning these factors is hardly feasible. To our knowledge, no study has yet investigated how changes in environmental macro-contexts affect the quality of psychedelic experiences.

In view of the abovementioned restrictions and changes in everyday life and their reported impact on mental health in general, we interpret the COVID-19 pandemic as such a global and profound macro-contextual change. Most groups of psychoactive substances like alcohol, opioids, sedatives and stimulants reliably induce pleasant transient mood states, e.g., feelings of euphoria and/or anxiolysis, that might be associated with maladaptive coping mechanisms and increased addiction potential in the face of the pandemic (9, 23). In contrast, psychedelics were expected to be less attractive as coping tools during a global public health crisis. Thus, we aimed to explore the effect of the COVID-19 pandemic on different aspects of (serotonergic) psychedelic use. Using an international, cross-sectional, internet-based survey during the early COVID-19 pandemic, we examined the effects of the pandemic on (1) the setting in which participants would use psychedelics, (2) the motives to use psychedelics, and (3) the quality of psychedelic experiences. Due to the sensitivity of psychedelic experiences to extra-pharmacological, contextual factors, it was expected that an increase of aversive environmental conditions during the COVID-19 pandemic would be associated with an increase in unpleasant and challenging psychedelic experiences.

## Methods

### Procedure

Study data were collected as part of the larger, anonymous, internet-based “Corona Drug Survey.” This cross-sectional survey investigated how psychoactive substance use was affected by the COVID-19 pandemic. The survey was online between April 30, 2020 and August 4, 2020 and available in five languages (English, German, Spanish, Italian, Korean). The study was approved by the Ethics Committee of the Charité—Universitätsmedizin Berlin, Germany (EA1/109/20).

#### Inclusion and Exclusion Criteria

Eligible participants had to be at least 18 years old, fluent in one of the five languages available, and had to have used at least one of the following substances in 2019 or 2020: alcohol, nicotine, cannabis, benzodiazepines, cocaine, amphetamine, ecstasy, psychedelics, dissociatives, opioids, gamma-hydroxybutyric acid (GHB)/gamma-butyrolactone (GBL), or new psychoactive substances (NPS). The present analysis included data of participants that reported to have taken serotonergic psychedelics (5-HT_2A_ receptor agonists). This section included the following substances: lysergic acid diethylamide (LSD), psilocybin, ayahuasca, N,N-dimethyltryptamine (DMT), 5-methoxy-DMT (5-MeO-DMT), mescaline, 2,5-dimethoxy-4-bromophenethylamine (2C-B), new psychedelics [e.g., 1-propionyl-LSD (1P-LSD)], or other (serotonergic) psychedelics. Data on other substances sometimes referred to as psychedelics [e.g., ketamine or 3,4-methylenedioxymethamphetamine (MDMA)], as well as data of non-psychedelic substances will be published elsewhere.

#### Recruitment of Participants

Participants were recruited via online postings and advertisements on various Internet websites (e.g., www.reddit.com), e-mail announcements, and articles in online magazines (e.g., “VICE”). Interested participants could inform themselves about the goals of the survey on a project landing page. If they wanted to start the survey, participants were redirected to the survey that was hosted on the secure online platform “SoSci Survey,” a German survey tool (www.soscisurvey.com) (24). Participants then filled out the questionnaire described below. If participants omitted questions of the survey, they were asked to answer the missing questions at the end of each page. However, participants could nevertheless choose to skip the missing items, which resulted in varying sample sizes across the items.

### Design of Online Survey

#### Demographics

Participants were asked for their age, gender, country of origin, level of education, work situation, housing situation (household size and number of children living in the same household), and frequency of psychedelic and other drug use.

#### Effects of the COVID-19 Pandemic on the Participants' Personal Lives

The survey included questions about how the COVID-19 pandemic affected the participants' personal lives. This section included questions about lifestyle restrictions associated with the pandemic, personal experiences of quarantine, changes in the work situation, changes in price and quality of psychedelic drugs, as well as the personal evaluation of the current situation (e.g., worries and concerns, as well as confidence to be able to cope with the situation). Furthermore, participants were asked about positive changes (e.g., new hobbies, more free time) associated with the pandemic using a multiple-choice list of options. Increase in psychopathological symptoms during the pandemic was measured with a modified version of the 9-item short version of the symptom check list (SCL-9) (25). Respondents were asked to directly indicate the change in severity of each symptom on a five-point Likert scale from “much less” to “much more.” Please note that not all versions represent published translations but were translated from German by our translators.

#### Setting and Motives to Use Psychedelics

Participants were asked how the dosage of psychedelics changed in the last 4 weeks compared to before the COVID-19 pandemic on a five-point Likert scale from “much lower” to “much higher.” For both periods separately [before the pandemic/ in the last 4 weeks (during the COVID-19 pandemic)] participants were asked to indicate the settings in which they had consumed, and their motives to consume using pre-set multiple-choice lists (see **Figures 2**, **3**).

#### Quality of Psychedelic Experiences

If participants reported to have taken psychedelics in the last 4 weeks (during the COVID-19 pandemic), they were presented with a list of 14 common phenomenological aspects of psychedelic experiences. They were asked to indicate on a five-point Likert scale ranging from “much less” to “much more” what kind of experiences they had in the last 4 weeks compared to before the COVID-19 pandemic (see **Figure 4**). A complete overview of items and questions from the survey is available in the supplement of this article.

### Analyses

Data quality was assessed and participants that showed indicators of poor data quality were excluded before data analysis: Data sets were excluded if participants did not answer at least one question regarding their experiences with psychedelics. Furthermore, data were excluded if participants choose mutually exclusive response options (e.g., indicating that they took psychedelics for the first time during the COVID-19 pandemic but simultaneously reporting substance use before the pandemic). Lastly, data sets were excluded if their relative speed index was above the recommended cut-off of 2.0 (26).

A detailed sample description was performed to verify macro-contextual changes (i.e., changes in the social, cultural, and economic milieu) associated with the COVID-19 pandemic during the last 4 weeks.

Changes in the micro-context (i.e., setting and motives) before and in the last 4 weeks (during the COVID-19 pandemic) were analyzed using McNemar's test for paired samples.

In participants that reported psychedelic use in the last 4 weeks (during the COVID-19 pandemic), changes in the quality of psychedelic experiences were analyzed using single-sample *t*-tests and changes were correlated with reported pandemic-related restrictions, concerns, and psychopathology using Spearman's rank correlation coefficient. Statistical significance was set to *p* < 0.05, Bonferroni-corrected for multiple comparisons.

Lastly, it was explored whether participants that had taken psychedelics during the pandemic differed from participants that had not taken psychedelics during the pandemic but before the pandemic. Based on data level, group comparisons were performed using *t*-tests, Mann-Whitney *U*-tests, or chi-squared tests. As these analyses were exploratory, no Bonferroni-correction was applied.

All statistical analyses were performed using PASW Statistics 18 (27).

## Results

### General Sample Description

Of the total number of 5,049 participants of the Corona Drug Survey, 1,588 (31.5%) reported the use of psychedelic drugs in 2019 or 2020. Of those, 213 datasets (13.4%) were excluded because of quality reasons, yielding a total sample size of *n* = 1,375 participants.

Participants from 53 different countries completed the survey. Most participants originated from Germany (29.0%), Mexico (17.6%), Colombia (12.6%), Argentina (6.3%), USA (5.5%), India (3.7%), UK (3.6%), Bolivia (3.8%), and Italy (2.5%). The questionnaire was completed in the following languages Spanish (43.3%), English (29.5%), German (24.4%), and Italian (2.8%). A detailed sample description is provided in Table 1.

**Table 1:** Sample description.

	**Before the pandemic**		**In the last 4 weeks (during pandemic)**				
	**User**		**User**		**Non-user**		***P* ^a^**
	***n***	**(%)**	***n***	**(%)**	***n***	**(%)**	
**Sample size**	1,332	(96.9)^b^	641	(46.6)^b^	734	(53.4)^b^	
**Age in years (range)**	26.6	(18-67)	26.6	(18-62)	26.4	(18-67)	0.574
**Gender**							0.417
Female	471	(35.4)	220	(34.3)	274	(37.3)	
Male	811	(60.9)	392	(61.2)	437	(59.5)	
Non-binary	33	(2.5)	20	(3.1)	15	(2.0)	
**Education (at time of survey)**							0.586
University degree	612	(46.0)	297	(46.3)	334	(41.9)	
Secondary school degree	561	(42.1)	273	(42.6)	308	(45.6)	
Vocational training	111	(8.3)	54	(8.4)	59	(8.0)	
No degree	48	(3.6)	17	(2.7)	33	(4.5)	
**Work situation (before COVID-19 pandemic)**							0.050
Employed	531	(39.8)	276	(43.1)	272	(37.1)	
Freelancer/Self-employed	271	(20.4)	126	(19.7)	150	(20.4)	
Temporary employment	83	(6.2)	34	(5.3)	50	(6.8)	
Seeking work	168	(12.6)	67	(10.5)	105	(14.3)	
Unpaid domestic work/ Parental leave	20	(1.5)	13	(2.0)	9	(1.3)	
Student employment	231	(17.3)	116	(18.1)	129	(17.6)	
Retired	15	(1.1)	5	(0.8)	10	(1.4)	
**Household size**							0.381
No other person	179	(13.4)	97	(15.1)	91	(12.4)	
1 other person	226	(17.0)	120	(18.7)	116	(15.8)	
≥2 other persons	927	(69.6)	424	(66.2)	527	(71.8)	
**Children living in household**							0.100
None	1,060	(79.6)	501	(78.2)	598	(81.5)	
1 child	147	(11.0)	70	(10.9)	78	(10.6)	
≥2 children	123	(9.3)	69	(10.8)	57	(7.8)	
**Psychedelic substance use**
LSD	1,147	(86.1)	445	(69.4)		
Psilocybin	739	(55.5)	205	(32.0)		
2C-B	324	(24.3)	85	(13.3)		
DMT	307	(23.0)	76	(11.9)		
New Psychedelics	248	(18.6)	66	(10.3)		
Mescaline	156	(11.5)	32	(5.0)		
Ayahuasca	117	(8.8)	21	(3.3)		
5-MeO-DMT	63	(4.7)	13	(2.0)		
Other	24	(1.8)	6	(0.9)		
**Other substances used in 2019 or 2020**							0.276
Alcohol	1,245	(93.5)	594	(92.7)	691	(94.1)	
Cannabis	1,244	(93.4)	592	(92.4)	691	(94.1)	
Nicotine	958	(71.9)	445	(69.4)	535	(72.9)	
MDMA	798	(59.9)	386	(60.2)	427	(58.2)	
Cocaine	727	(54.6)	334	(52.1)	410	(55.9)	
Amphetamines/ Methamphetamines	513	(38.5)	255	(39.8)	273	(37.2)	
Dissociatives	484	(36.3)	254	(39.6)	244	(33.2)	
Benzodiazepines	332	(24.9)	172	(26.8)	167	(22.8)	
Opioids	164	(12.3)	87	(13.6)	81	(11.0)	
New Psychoactive substances	160	(12.0)	86	(13.4)	77	(10.5)	
GHB/GBL	101	(7.6)	47	(7.3)	58	(7.9)	

*User/Non-User refers to use of psychedelics in the specified time periods*.

*Unless otherwise stated, percentages refer to the respective sub-sample*.

a *Refers to comparison between users and non-users*,

b *percentages from total sample size of n = 1,375*.

The majority of participants (*n* = 1,332; 96.9%) reported that they had used psychedelics before the COVID-19 pandemic, 641 participants (46.6%) reported that they had taken psychedelics in the last 4 weeks (during the pandemic), 43 participants (3.1%) reported that they had used psychedelics for the first time since the beginning of the COVID-19 pandemic. The three psychedelic substances most used before the pandemic were LSD, psilocybin, and 2C-B. Most of the participants reported that they also consumed other substances in 2019 or 2020 (see Table 1). Frequencies of psychedelic substance use are presented in Table 2. For *n* = 1,332 participants that had used psychedelics already before the pandemic, median frequency of monthly use for the participants' most commonly used psychedelic changed from 0.30 times per 4 weeks before the pandemic (estimated from yearly median 3.95) to 0.91 times in the last 4 weeks during the pandemic.

**Table 2:** Frequencies of psychedelic substance use.

**Before the pandemic** **(per year)**			**In the last 4 weeks** **(during pandemic)**		
	***n***	**(%)**		***n***	**(%)**
No use^a^	43	(3.1)	No use	734	(53.4)
1-4 days	678	(49.3)	1 day	291	(21.2)
5-14 days	293	(21.3)	2-4 days	239	(17.4)
15-24 days	119	(8.7)	5-9 days	61	(4.4)
25-49 days	72	(5.2)	10-14 days	21	(1.5)
50 days or more	170	(12.4)	15 days or more	29	(2.1)

*Percentages from total sample size of n = 1,375 indicate frequency of substance use for the psychedelic substance that participants reported they had used most often*.

a *n = 43 participants reported first time use of psychedelics during the last 4 weeks*.

### Effects of the COVID-19 Pandemic on the Participants' Personal Lives (Macro-Contextual Changes)

**Figure 1:**
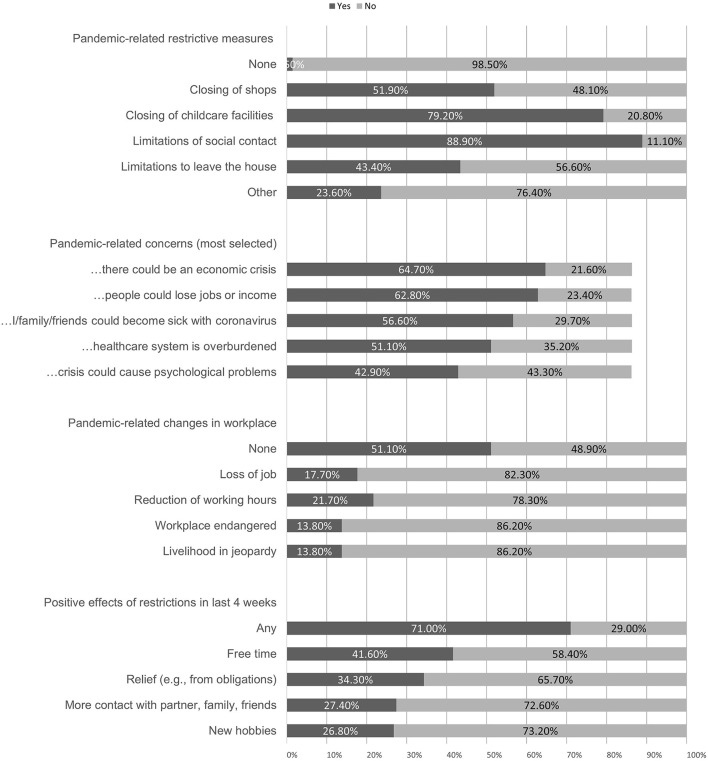
COVID-19 pandemic related changes. Pandemic-related concerns were only asked of those participants who had indicated that they were at least somewhat concerned about developments around the coronavirus in the last 4 weeks (86.2%).

Almost all participants (*n* = 1,354; 98.5%) stated that they had faced some form of restrictive measures in their surroundings due to the pandemic. Most participants (67.0%) reported to adhere to these measures rather well or very well. Only few people reported to adhere to the measures not at all (1.5%) or not very well (5.5%). A little more than half of the participants (57.4%) indicated that they had been in quarantine at least temporarily during the last 4 weeks; 16.2% had experienced cold symptoms themselves during the last 4 weeks; 3.9% had already been tested for COVID-19, and 0.2% had received a positive test result. Figure 1 gives an overview of pandemic-related changes reported by the subjects.

Most participants (65.1%) felt that they had restricted their lifestyle in the last 4 weeks compared to before the pandemic significantly; 86.6% left their house less frequently; 71.8% felt at least partially socially isolated. Most participants (86.2%) were at least somewhat concerned about the COVID-19 pandemic, 8.1% were very significantly concerned. Most common concerns are presented in Figure 1. Half of the participants (54.8%) were a little or very confident that they would be able to cope with the current crisis by themselves, 18.6% were not or barely confident they could cope with it. Participants reported a slight increase in psychopathological symptoms as measured with the SCL-9 (mean of 3.35 on a scale from 1 to 5 from “much less” to “much more”).

A little more than half of the participants (56.7%) reported that the price for psychedelics was unchanged, 2.3% reported it was lower, 27.7% reported it was higher. Two third of the participants (68.1%) reported that the quality for psychedelics was unchanged, 12.8% reported it was lower, 5.3% reported it was higher.

### Settings and Motives to Use Psychedelics (Micro-Contextual Changes)

A total number of 641 participants (46.6%) reported psychedelic substance use during the pandemic. Most of them (93.3%) stated that they also used psychedelics before the pandemic. There was no change in the top three substances used before/during the pandemic: Most of the participants reported experiences with LSD, followed by psilocybin, and 2-CB (see Table 1). Half of the participants (51.3%) reported no change of their dosage, 20.6% reported it was lower, 20.5% reported a higher dosage.

#### Settings

**Figure 2:**
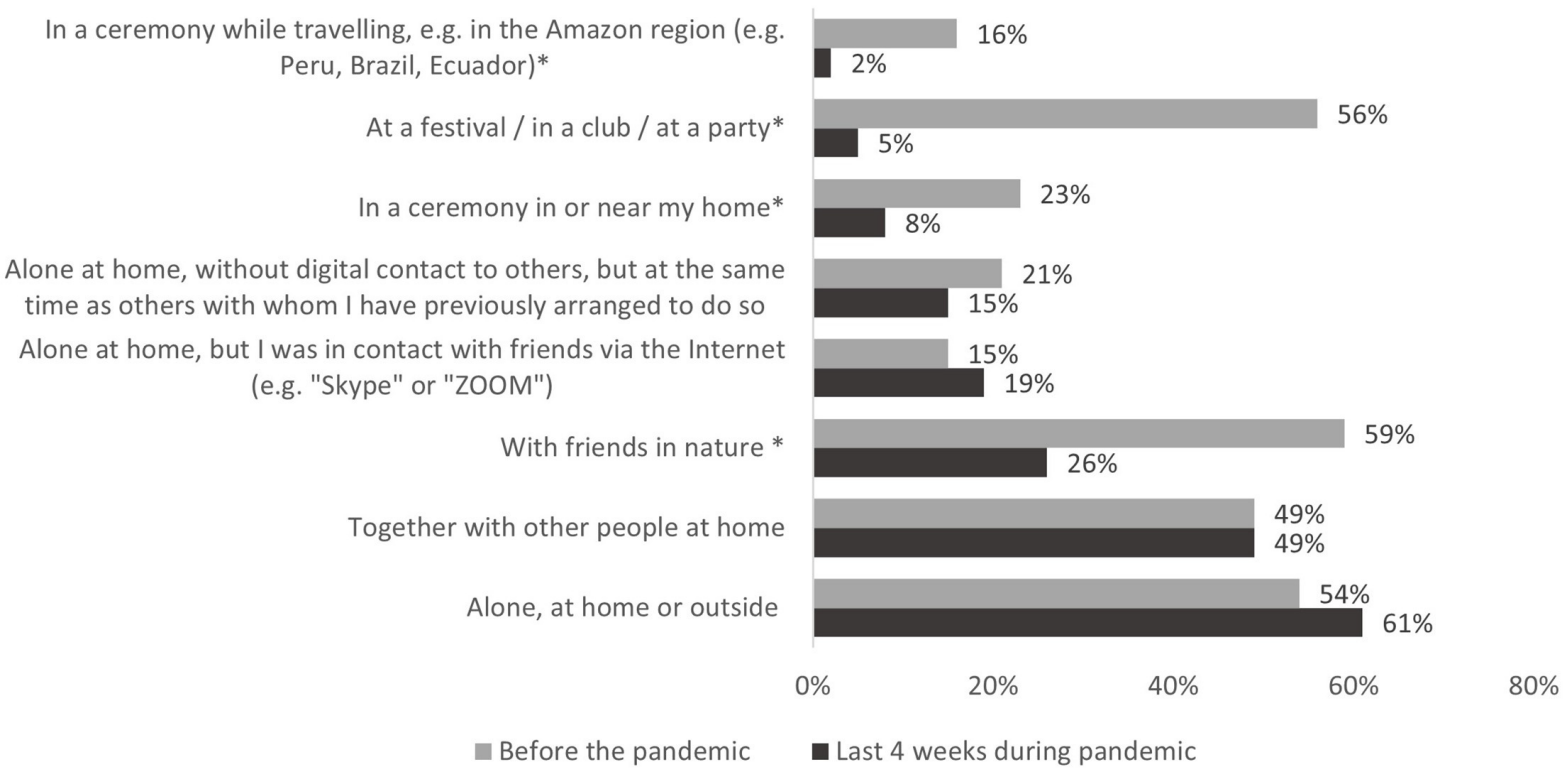
Setting during psychedelic substance use. *Indicates statistically significant difference in the comparison before the pandemic vs. last 4 weeks during the pandemic (McNemar test, *p* < 0.05, Bonferroni-corrected).

Figure 2 displays the settings in which participants reported use of psychedelics before/in the last 4 weeks (during the COVID-19 pandemic). During the last 4 weeks of the pandemic, most participants reported having taken psychedelics (1) alone, at home or outside or (2) together with other people at home. There was a significant decline in all settings that were outside the participants' home (see Figure 2). This included ceremonies and parties but also the use of psychedelics with friends in nature, which had been the most frequently reported setting before the pandemic.

#### Motives to Use Psychedelics

**Figure 3:**
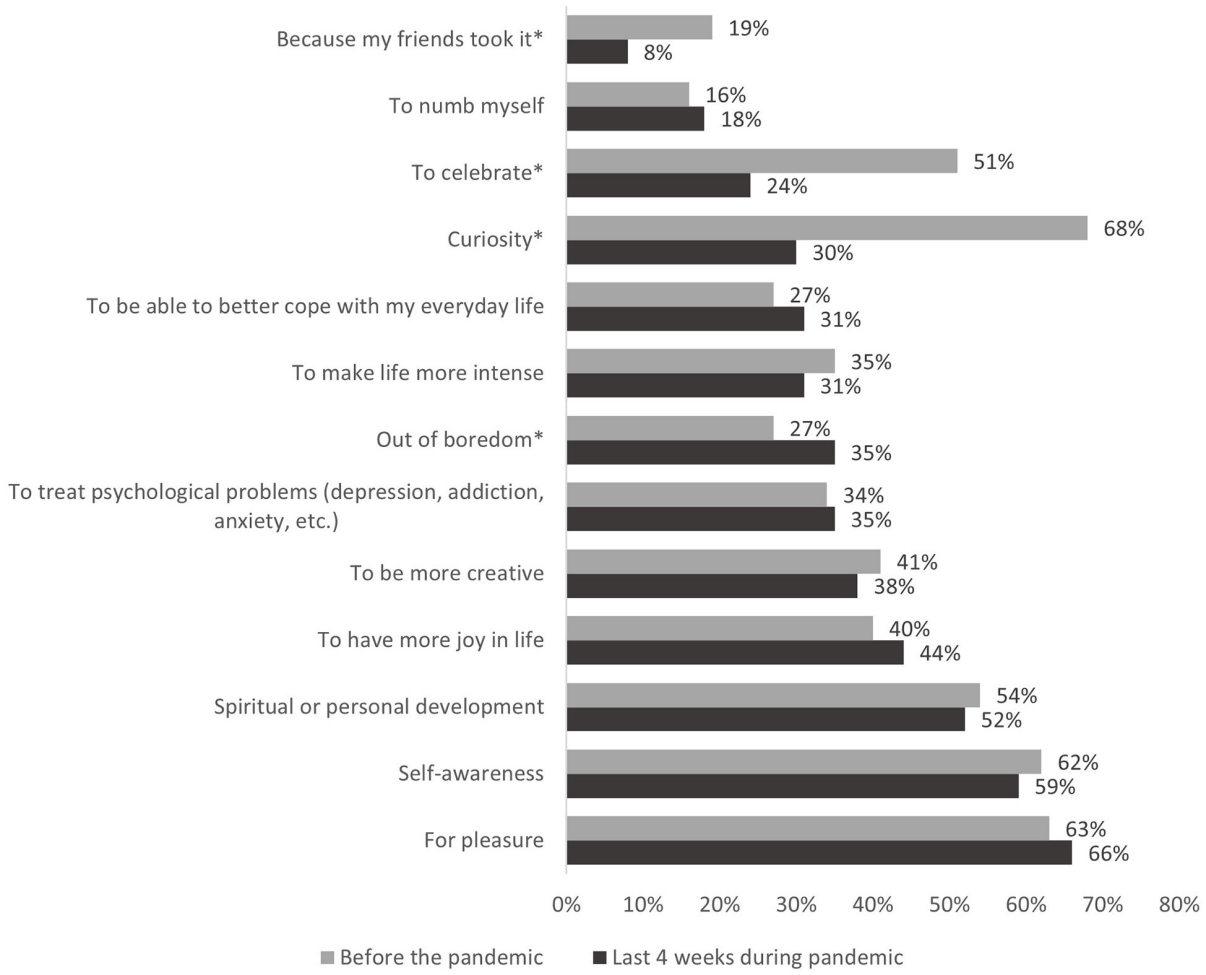
Motives to use psychedelic substances. *Indicates statistically significant difference in the comparison before the pandemic vs. last 4 weeks during the pandemic (McNemar test, *p* < 0.05, Bonferroni-corrected).

Figure 3 displays the reasons why participants reported having used psychedelics before/in the last 4 weeks (during the COVID-19 pandemic). The top three reasons why participants chose to use psychedelic substances in the last 4 weeks (during the pandemic) were (1) pleasure (2) self-awareness (3) spiritual or personal development. Those reasons were largely identical with the top three reasons before the pandemic, except that during the COVID-19 pandemic participants consumed considerably less often out of curiosity. Furthermore, participants less often reported consuming because friends took it or to celebrate and more often out of boredom compared to before the pandemic.

### Quality of Psychedelic Experiences

**Figure 4:**
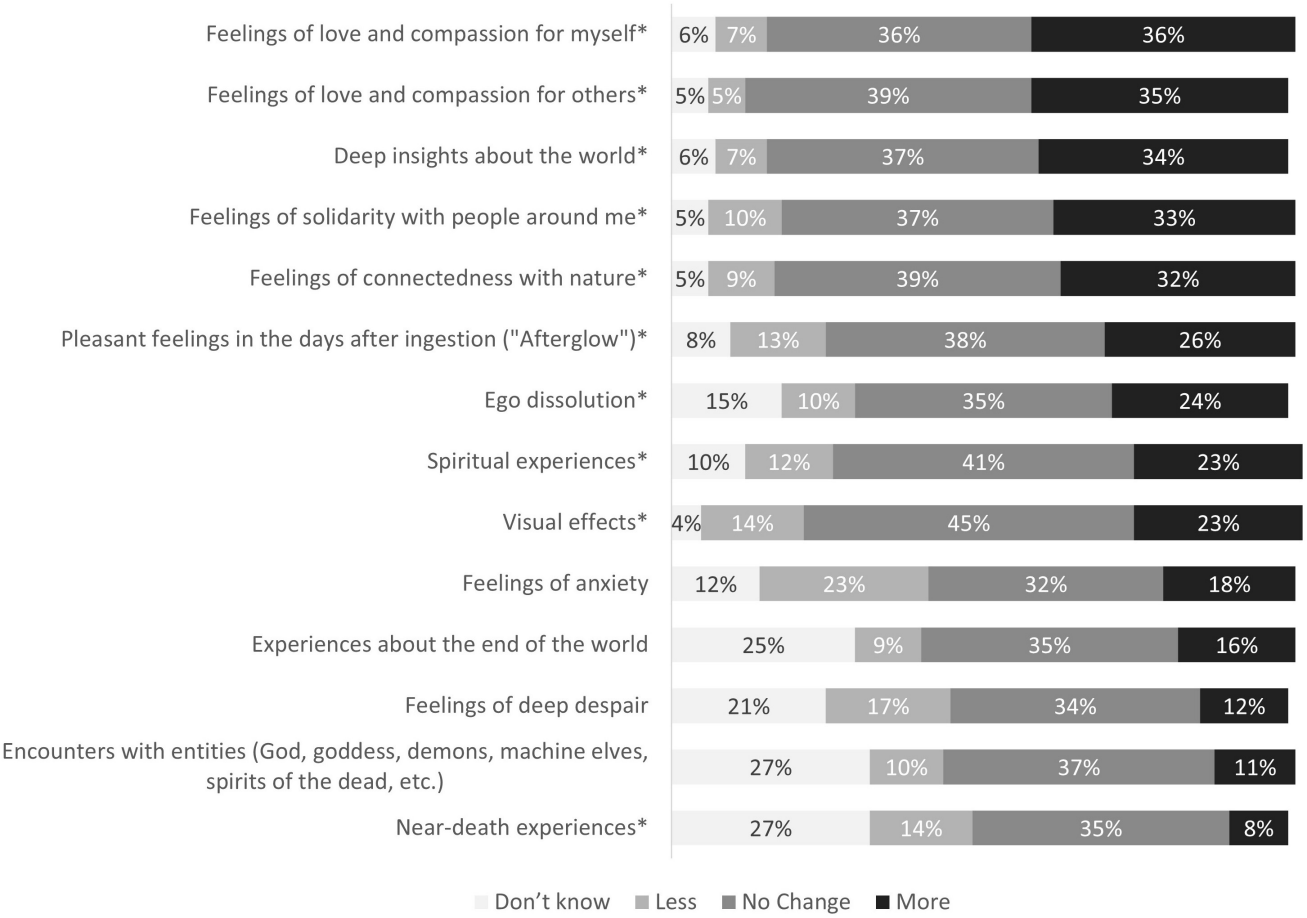
Psychedelic experiences in the last 4 weeks compared to before the COVID-19 pandemic. Numbers indicate percentage of *n* = 598 participants that reported to have used psychedelics during the COVID-19 pandemic and that also had used psychedelics before the pandemic. For each item, percentages not totaling 100% signify missing values. *Indicates statistically significant difference at *p* < 0.05, Bonferroni-corrected.

Figure 4 displays 14 common phenomenological aspects of psychedelic experiences reported by participants comparing their experiences before the pandemic vs. in the last 4 weeks (during the COVID-19 pandemic). Approximately one third of the participants did not report changes in their experiences. Among the other participants, both increases and decreases of certain experiences were reported. A significant increase during compared to before the pandemic was observed in the following areas: feelings of love and compassion for myself, *t*_(474)_ = 12.23, *p* < 0.001; feelings of love and compassion for others, *t*_(477)_ = 12.08, *p* < 0.001; deep insights about the world, *t*_(472)_ = 11.24, *p* < 0.001; feelings of connectedness with nature, *t*_(479)_ = 9.09, *p* < 0.001; feelings of solidarity with people around me, *t*_(482)_ = 8.08, *p* < 0.001; ego dissolution, *t*_(415)_ = 5.80, *p* < 0.001; pleasant feelings in the days after ingestion (“Afterglow”), *t*_(460)_ = 4.84, *p* < 0.001; spiritual experiences, *t*_(452)_ = 4.62, *p* < 0.001; visual effects, *t*_(485)_ = 3.77, *p* < 0.001. A significant decrease was observed in near-death experiences, *t*_(346)_ = −3.64, *p* < 0.001.

No significant changes (after Bonferroni correction) were observed in the following areas: feelings of anxiety, *t*_(438)_ = −2.72, *p* = 0.007; feelings of deep despair, *t*_(380)_ = −2.88, *p* = 0.004; experiences about the end of the world, *t*_(358)_ = 1.59, *p* = 0.113; encounters with entities (God, goddess, demons, machine elves, spirits of the dead, etc.), *t*_(342)_ = 0.12, *p* = 0.908.

There was a positive correlation between increased psychopathological symptoms as measured with items of the SCL-9 and feelings of anxiety, r_s_ = 0.26, *p* < 0.001, and deep despair, r_s_ = 0.22, *p* < 0.001, during psychedelic experiences. Correlations between changes in the quality of psychedelic experiences and restrictions, concerns, or changes in the dosage related to the pandemic situation were very weak and negligible (all r_s_ <0.2).

We furthermore explored whether positive changes in the quality of psychedelic experiences were associated with positive effects of restrictions reported by the participant (as described in Figure 1). Participants that reported that the initial restrictions had any positive effect for them showed a significantly greater change in the quality of psychedelic experiences (in the direction described above) than participants that did not report any positive effect of restrictions. This was true for all abovementioned effects except for feelings of connectedness with nature, ego dissolution, visual effects, and near death-experiences. Nevertheless, albeit smaller, the effects in the three categories feelings of love and compassion for myself, feelings of love and compassion for others, and deep insights about the world were still significant in the group of participants that did not report positive effects of restrictions.

Regarding the assessment of the usefulness of psychedelics in dealing with different aspects of the pandemic, the following data were observed: 26.8% of participants that used psychedelics in the last 4 weeks during the pandemic did not agree that psychedelics would help them to deal better with the corona crisis, 41.0% agreed slightly or moderately, 25.9% agreed very much or absolutely. Half of the participants (49.6%) did not think that psychedelics would help them to make sense of the corona crisis, 29.8% agreed slightly or moderately, 14.5% agreed very much or absolutely. Furthermore, half of the participants (49.5%) felt that consuming psychedelics did not change their ability to cope with social distancing in the last 4 weeks, while 4.6% reported to cope worse and 39.6% reported to cope better with social distancing by taking psychedelics.

### Comparison of Participants That Used Psychedelics During the Pandemic and Participants That Used Psychedelics Before the Pandemic, but Not During the Pandemic

Participants that used psychedelics in the last 4 weeks during the pandemic (users) and participants that used psychedelics before the pandemic but not in the last 4 weeks (non-users) did not significantly differ in age, gender, education, work situation, household size, or number of children living in the household (see Table 1). Also there were no differences in the frequency of quarantine, χ^2^_(1)_ = 2.77, *p* = 0.096, cold symptoms, χ^2^_(1)_ = 0.22, *p* = 0.637, or whether professional life changed since the beginning of the crisis, χ^2^_(5)_ = 6.09, *p* = 0.297. Groups did not differ in whether they felt socially isolated, U = 227,908, *p* = 0.302, or concerning how confident they were about coping with the current crisis on their own, U = 224,133, *p* = 0.130, or the general frequency with which they reported that the initial restrictions also had positive effects, χ^2^_(1)_ = 1.39, *p* = 0.238. Furthermore, there were no differences between the two groups concerning changes of psychopathological symptoms during the pandemic as measured with the SCL-9, *t*_(1339)_ = 0.04, *p* = 0.969.

However, people that did use psychedelics felt significantly less restricted in their lifestyle, U = 205,314, *p* < 0.001, less concerned, U = 218,818, *p* = 0.014, and left their home more frequently, U = 215,044, *p* = 0.002, than participants that did not use psychedelics during the pandemic. Furthermore, participants that used psychedelics during the pandemic reported a higher frequency of psychedelic substance use before the pandemic when compared to non-users, U = 185,643, *p* < 0.001. There was no difference between the groups in the number of other psychoactive substances used in 2019 or 2020, U = 227,319, *p* = 0.276.

## Discussion

In the present study we explored how the social and cultural macro-contextual changes of the early COVID-19 pandemic affected the setting, motives to use psychedelics, and quality of psychedelic experiences. Our data showed that, during the pandemic, participants used psychedelics significantly less often in settings that were outside their home compared to before the pandemic. Top motives to use psychedelics included pleasure, self-awareness, and spiritual or personal development. Motives were largely comparable before and during the pandemic, but participants consumed less out of curiosity, to celebrate, or because friends took it, and more out of boredom. A significant increase in positively connoted, often pro-social experiences, e.g., feelings of love, compassion, and connectedness but not in challenging experiences was observed. Two thirds of participants who used psychedelics during the pandemic claimed that psychedelics would have at least slightly helped them to better deal with the corona crisis.

As expected, almost all participants in our sample experienced relevant and serious changes in their socio-cultural environments due to the COVID-19 pandemic. Almost all participants faced some form of measures to contain the spread of the virus, most felt restricted in their lifestyle, isolated, or concerned about the current situation, and reported increased psychopathology. These data are in line with previous reports on the extensive impact of the COVID-19 pandemic in daily life (2) and studies that observed declined mental health outcomes during the pandemic (4–6, 28). Because it was previously suggested that the COVID-19 pandemic could affect the trade business for drugs (29), we also assessed changes in quality and prices of psychedelic substances. In this survey, quality of psychedelics was reported to be largely the same, with few people reporting increases or decreases of quality. One fourth of the subjects reported that the price for psychedelics had increased. Data are in line with observations of the European Monitoring Centre for Drugs and Drug Addiction and Europol that observed that the COVID-19 pandemic temporarily disrupted the drug market resulting in higher prices for some drugs (7).

In accordance with the restrictions of social contacts imposed by COVID-19 measures, most participants reported having used psychedelics during the pandemic alone, at home or outside. There was a steep decline in all settings that were outside the participants' homes and that involved other people (e.g., ceremonies, with friends in nature). Participants also reported that during the pandemic they had taken psychedelics considerably less often at parties and festivals.

The most common motives for using psychedelics during the pandemic were largely identical with the motives for using psychedelics before the pandemic and included pleasure, self-awareness, and spiritual or personal development. Consistent with the pandemic-related changes of the setting, participants reported having consumed less often because friends took it or to celebrate. Furthermore, participants reported having consumed less often out of curiosity and more out of boredom. The increase in substance use out of boredom is a frequent finding during the COVID-19 pandemic and has been described for other substances, like alcohol, cigarettes, and cannabis (30, 31).

To our surprise, and despite macro-contextual changes of a globally present public health-crisis that negatively affected large parts of public and private life, most participants did not report an increase in challenging psychedelic experiences when compared to the time before the pandemic: Feelings of anxiety, feelings of deep despair, and experiences about the end of the world did not significantly differ during compared to before the COVID-19 pandemic. Near death-experiences were even significantly reduced. On the contrary, an increase in feelings of love and compassion for myself, feelings of love and compassion for others, feelings of connectedness with nature, feelings of solidarity with people around me, and deep insights about the world during psychedelic experiences, was reported by up to one third of participants, along with an increase in ego dissolution, pleasant feelings in the days after ingestion (“afterglow”), spiritual experiences, and visual effects. In the following, we explore some ideas that might explain the findings.

The term psychedelics has long been coined to address the “mind-manifesting” properties of those substances which have been suggested to reveal “hidden” aspects of mind and soul, in order to explore oneself and deepen processes of introspection (32). Accordingly, in times of a global public health crisis, measures of social distancing and profound feelings of disconnectedness, serotonergic psychedelics might have sensitized users to pro-social attitudes and revealed a desire to reconnect and interact with others, while feeling more empathetic toward themselves and others.

Furthermore, it was expected that the pandemic would negatively affect the individual micro-context during substance use. Our data suggest, however, that some macro-contextual aspects of the pandemic may paradoxically have had beneficial effects on individual circumstances of substance use. Although parties and festivals might be described as supportive and positive settings for many users, environmental aspects such as music, lightning, and social interactions might be associated with more unpredictable, overwhelming, and uncontrollable situations. Less controlled settings, in turn, have been identified as a potential risk factor for challenging experiences, as feelings of insecurity or anxiety may quickly escalate (13). Thus, reduced use of psychedelic substances in less controlled settings could have positively modulated the quality of psychedelic experiences. Further evidence for this explanation came from an exploratory *post-hoc* analysis, according to which the observed changes in psychedelic experiences occurred primarily in individuals who reported that the restrictions also brought positive effects (e.g., more free time, or relief from obligations). Thus, more positively connoted changes in psychedelic experiences could possibly be related to the omission of higher-risk consumption sites and/or other beneficial changes in the (micro-) set or setting (e.g., no time pressure, less stress through fewer obligations).

Another reason for heightened positively connoted experiences might be a strong self-selection bias among psychedelic substance users encouraged by the onset of the pandemic. This possibility seems plausible since only half of the participants that had used psychedelic drugs in the year before the pandemic also used psychedelics in the last 4 weeks during the pandemic. As psychedelics are well-known to be context-sensitive (19, 33), certain users might simply have stopped using psychedelics, in order to prevent anticipated “bad trips.” Indeed, several differences between users and non-users were observed. For instance, participants that used psychedelics during the pandemic felt less restricted, less concerned, left their home more frequently during the pandemic, and had used psychedelics before the pandemic at a higher frequency than participants that had used psychedelics in the year before the pandemic but not in the last 4 weeks during the pandemic. Thus, the increase in positive experiences in our sample might partially be caused by the abstinence of more restricted and less experienced users that possibly would have been more vulnerable to challenging experiences. Since this survey was cross-sectional, it cannot be ruled out, however, that the decreased perception of restrictions was not a cause but a consequence of psychedelic substance use.

Intensified experiences could also have been caused by higher doses of psychedelics used during compared to before the pandemic. However, this seems unlikely as not all qualities were equally intensified, and instead a selective pattern of increases and decreases in different experiential qualities was observed. In addition, no significant correlations between the changes in psychedelic experiences and changes in dosages reported by participants were observed.

Taken together, regarding the quality of psychedelic experiences, the observations of the present survey suggest that it may be too simplistic to infer individual micro-contexts from environmental macro-contexts. While the pandemic situation and associated restrictions of lifestyle were present in most participants' lives, it is possible that macro-contextual changes on a global level did not necessarily have a direct negative impact on the individual set and setting during psychedelic substance use. On the contrary, some aspects of the pandemic may paradoxically have positively affected individual contexts of substance use. Therefore, more complex interactions between macro- and micro-contexts must be considered. To our knowledge, there is not yet any model available that describes how different contextual levels interact in their impact on psychedelic experiences, which would be beneficial in predicting individual psychedelic experiences more accurately. The importance of integrating larger-scale environmental aspects in models that predict psychedelic experiences was also suggested in a framework presented by Eisner. She suggested that the concept of “set and setting” should be extended to include another feature called “matrix” and should reflect “the environment surrounding the subject before and after the session, and the larger environment to which the subject returns” [(34), p. 214]. A helpful basis to conceptualize different contextual systems in future research may be derived from existing frameworks, such as the bioecological system theory (35), which emphasizes not only different contextual levels but also provides a conceptualization of possible interactions between these levels.

More than half of the participants that used psychedelics in the last 4 weeks during the pandemic (66.9%) at least slightly agreed that psychedelics would help them to better deal with the corona crisis. This estimate was much higher than for many other substances of the parent-survey, e.g., alcohol (33.9%) and “party drugs” (i.e., amphetamine, methamphetamine, MDMA, GHB/GBL, ketamine and dextromethorphan) (16.4-35.6 %), and slightly below the rating of cannabis (74.4%) (unpublished data). Importantly, these data reflect a subjective evaluation of benefits by the participants. Even though many users considered psychedelics as helpful to deal with the pandemic, no conclusions can be drawn on whether this coping was functional (e.g., supporting a healthy adaption to the pandemic situation in everyday life by promoting problem-focused coping strategies) or dysfunctional (e.g., supporting denial and behavioral or mental disengagement). Traditionally, substance-related coping strategies are often regarded as dysfunctional. However, it has previously been argued that psychedelics could serve as “agents to enhance the perception of meaning” [(36), p. 1], and this idea has especially been emphasized with regard to psychedelic-assisted therapy for the treatment of existential anxiety and depression in patients suffering from life-threating illness [(37, 38), for a review see (39)]. In line with this, 44.3% of the participants agreed that psychedelics helped them at least slightly to make sense of the corona crisis. However, most data on beneficial effects of psychedelics originate from highly controlled clinical trials and cannot necessarily be generalized to recreational settings. Two recent papers investigated associations between lifetime psychedelic use and mental health outcomes during the COVID-19 pandemic in anonymous online surveys during the early COVID-19 pandemic (40, 41). Cavanna et al. (40) assessed lifetime use of psychedelics and other psychoactive drugs, as well as measures of personality, affect, well-being, and resilience in participants from Argentina. No associations between lifetime use of psychedelics and impaired mental health were observed. Instead, certain psychedelics were linked to indicators of improved mental health (e.g., higher score of positive affect). Révész et al. (41) assessed associations between lifetime use of psychedelics and psychological distress, peritraumatic stress, social support, and psychopathological symptoms in participants from different countries (mainly Spain, Brazil, and USA). Interestingly, they observed that psychedelic substance users reported less psychological distress during the pandemic than non-users and that effects were more pronounced for regular compared to occasional users. However, because of the correlational design of both studies, no conclusion on causality can be drawn on whether psychedelics serve as a protective factor or whether psychedelics are simply used by more resilient people. Therefore, it cannot be concluded from the current data that the use of psychedelics during the crisis would have a beneficial effect on mental health. Even if most psychedelics exhibit low toxicity and little addictive potential, the uncontrolled use of psychedelics might contribute to the occurrence of mental health problems (13). Findings in this paper should therefore by no means encourage psychedelic substance use. Although many participants reported an increase in positively connoted psychedelic experiences, around 10-20% of the participants reported more challenging or less positive psychedelic experiences during the pandemic. The increase in anxiety and despair during the experience was associated with a general increase in psychopathological symptoms during the pandemic, but not with the level of restrictions reported. This could indicate that the internal processing of changes associated with the pandemic is more relevant for shaping psychedelic experiences than the extent of the objective restrictions. Since this survey was cross-sectional, it cannot be ruled out, however, that increased psychopathological symptoms were not a cause of challenging experiences, but that challenging experiences negatively affected mental well-being of participants, leading to increased levels of psychopathology.

Another aspect of the survey concerned the frequency of substance use. We reported that the median frequency of substance use for the psychedelic substance that participants reported to have used most often was with 0.94 times during the last 4 weeks higher than the estimated frequency of 0.30 times per 4 weeks before the pandemic. However, those numbers should be interpreted with great caution. The monthly psychedelic use before the pandemic was estimated based on the pre-pandemic median annual consumption. However, psychedelics differ from other substances in that the frequency of use is often irregular and less than once per month and it cannot be assumed that consumption is evenly distributed throughout the year. The comparison of a single month with an annual average can therefore easily be distorted. In order to validly assess whether the COVID-19 pandemic is associated with changes in the frequency of psychedelic use, longer observation periods will be needed.

### Limitations

The study has several limitations. Recruitment for anonymous online surveys is always prone to a biased sample selection that is not fully representative of the general population investigated, in this case, users of serotonergic psychedelics. Our sample consisted of relatively young, highly-educated participants that reported many experiences with other psychoactive substances. Results may not be generalizable to other groups of users.

As mentioned above, data collection was cross-sectional. The description of associations between pandemic-related measures, as well as changes in psychedelic experiences is therefore correlational and no conclusion on causality is possible.

Furthermore, pandemic-related restrictions and changes in psychedelic experiences were assessed using pre-set multiple-choice categories. It is possible that these categories did not completely cover all individual experiences perceived as personally significant by the respondent.

The survey period of the Corona Drug Survey coincided in many countries with the beginning or peak of the first pandemic wave, in which public knowledge of the virus as well as its consequences were still very limited. It is possible that results of this early phase cannot be generalized to later stages of the pandemic.

## Conclusion

We observed that the early phase of the COVID-19 pandemic was associated with changes in the use of serotonergic psychedelics, including the settings in which people use psychedelics, the motives of usage, and the subjective quality of psychedelic experiences. Surprisingly, those changes were not necessarily adverse in nature, with up to one third of participants reporting an increase in positively connoted psychedelic experiences, including increased prosocial feelings of love, solidarity, and connectedness. Two thirds of participants who used psychedelics during the pandemic claimed that psychedelics would have at least slightly helped them to better deal with the corona crisis. Possible explanations include an interaction of environmental macro- and individual micro-contexts during the pandemic which paradoxically may have positively affected the quality of psychedelic experiences (e.g., by reducing the use in more uncontrolled recreational settings or by encouraging a strong self-selection of substance users due to the expectation of “bad trips”). Increased pro-social feelings under psychedelics might reflect a desire for social interactions in times of social distancing and pandemic-related stress and anxiety.

## Data Availability Statement

The raw data supporting the conclusions of this article will be made available by the authors, without undue reservation.

## Ethics Statement

The studies involving human participants were reviewed and approved by the Ethics Committee of the Charité—Universitätsmedizin Berlin.

## Author Contributions

TM, IM, CM, SG, and RE contributed to conception and design of the study. SR and RM set up the online survey and website. DM, RG, AR, DJ, and MS revised the different language versions of the survey generated by machine translation. RE performed the statistical analysis and wrote the first draft of the manuscript. All authors contributed to manuscript revision, read, and approved the submitted version.

## Conflict of Interest

The authors declare that the research was conducted in the absence of any commercial or financial relationships that could be construed as a potential conflict of interest.

## Publisher's Note

All claims expressed in this article are solely those of the authors and do not necessarily represent those of their affiliated organizations, or those of the publisher, the editors and the reviewers. Any product that may be evaluated in this article, or claim that may be made by its manufacturer, is not guaranteed or endorsed by the publisher.
